# Baropodometry on women suffering from chronic pelvic pain - a cross-sectional study

**DOI:** 10.1186/1472-6874-11-51

**Published:** 2011-11-17

**Authors:** Carolina W Kaercher, Vanessa K Genro, Carlos A Souza, Mariane Alfonsin, Greice Berton, João S Cunha Filho

**Affiliations:** 1 Programa de Pós-Graduação em Medicina: Ciências Médicas - Universidade Federal do Rio Grande do Sul - Brasil; 2 Serviço de Ginecologia e Obstetrícia - Hospital de Clínicas de Porto Alegre - Brasil; 3 INSEMINE - Human Reproduction Center, Porto Alegre, Brasil

**Keywords:** baropodometry, chronic pelvic pain, endometriosis, postural abnormalities

## Abstract

**Background:**

Previous studies have associated chronic pelvic pain with a stereotyped pattern of movement and posture, lack of normal body sensations, a characteristic pain distribution. We aimed at evaluating if these postural changes are detectable in baropodometry results in patients with chronic pelvic pain.

**Methods:**

We performed a prospective study in a university hospital. We selected 32 patients suffering from chronic pelvic pain (study group) and 30 women without this pathology (regular gynecological work out - control group). Pain scores and baropodometric analysis were performed.

**Results:**

As expected, study group presented higher pain scores than control group. Study and control groups presented similar averages for the maximum pressures to the left and right soles as well as soles supports in the forefeet and hind feet. Women suffering from chronic pelvic pain did not present differences in baropodometric analysis when compared to healthy controls.

**Conclusions:**

This data demonstrates that postural abnormalities resulting from CPP could not be demonstrated by baropodometric evaluation. Other postural measures should be addressed to evaluate pelvic pain patients.

## Background

Chronic Pelvic Pain (CPP) is a major health problem [1], and it can be defined as a nonmalignant pain perceived in structures related to the pelvis; constant or recurring over a period of 6 months. In some cases it might be associated with negative cognitive, behavioral and social consequences [2]. Prevalence of CPP in the female population has been suggested to be 3.8% [3], however it can reach 40% in infertility patients [4]. CPP is a significant symptom in reproductive age women [5], with a direct impact on their marital, social and professional life [1,6]. Several papers with different methodological characteristics have shown an association of CPP with a negative impact on personal activities [7,8]. CPP accounts for approximately 40% of laparoscopies and 10 to 15% of hysterectomies [9]. It has been estimated that women who suffer from CPP take approximately three times more medication when compared to women without pain [5]. Additionally, pelvic pain is a cause of absenteeism from work in 15% of cases, being associated to reduced productivity and limitation of home activities [6,10]. An association was demonstrated between CPP and altered orthostatic position [11].

Studies have associated CPP with a stereotyped pattern of movement and posture changes, pain distribution, lack of normal body sensations and control [5,11]. There is evidence in literature that women who suffer from CPP present postural abnormalities, which may play an important role to increase morbidity and a difficulty to treat those patients. Some authors concluded that patients suffering from CPP presented standard characteristics when in some positions that could be called "pelvic pain protection pattern", therefore the posture of these patients should be inherently unstable and this pattern, considered as a means of overcoming used as a balance strategy [5,12]. Therefore, it is clear that CPP is a complex disease that diagnosis and treatment goes beyond the scope of gynecology because it may be associated with an altered muscle and postural pattern and could benefit from a multidisciplinary approach to decrease levels of pain [5,13,14]. Indeed, authors have demonstrated that standardized Mensendieck test could be helpful to CPP management. This test evaluates posture, movement, gait, sitting posture and respiration of CPP patients [15].

Baropodometric analysis allows the evaluation of dysfunctions of the feet. The principle is to map the pressure in each segment of the plantar surface using force platforms which, indirectly, indicates important postural abnormalities [16]. Hence, baropodometry could be used as an instrument to measure and evaluate the effect of pelvic pain in those patients: skeletal, muscular and, consequently, postural changes. This method is essential to understand the importance of plantar proprioreceptors and adoption of an modified orthostatic position which could result in an erratic postural adaptation secondary to CPP [17].

Considering the association of CPP and postural abnormalities we decided to investigate, for the first time, if the postural changes related to CPP are detectable by baropodometry results in patients with CPP.

## Methods

### Design and patients

We performed a cross sectional study with a sample of 62 female patients aged between 18 and 45 years submitted to gynecologic evaluation between June 2005 and May 2007. Patients in use of use of regular hormonal or analgesic medication in the previous six months were excluded from our sample. We were able to select 32 patients suffering from CPP (study group) and 30 women without CPP (regular gynecological work out - control group). We defined CPP as pain perceived in structures related to the pelvis; constant or recurring over a period of 6 months, as previously used [12,18,19]. The cause of CPP in the study group consisted in: 22 (68.7%) patients presenting endometriosis diagnosed through video laparoscopy [20], 1 (3.1%) patient presented irritable bowel syndrome [21] and 9 (28.2%) were under investigation for suspected endometriosis. Control group was sequentially selected from patients submitted to tubal ligature. The research was approved by local ethic committee (Grupo de Pesquisa e Pós-Graduação, Hospital de Clínicas de Porto Alegre) and all the patients signed a consent form.

### Protocol and evaluation

Baropodometry provides qualitative data of weight distribution in the segments of hindfoot, midfoot and forefoot and of displacements of the center of pressure. baropodometry in order to investigate the relationship which the body has with the ground during walking, jumping, or while maintaining a standing posture, in a way which is possible to obtain data pertinent to postural oscillation, ground contact area of the feet, peak contact pressure, and various other variables. It is known that such variables are useful in detecting functional alterations that may point out certain risk factors, such as instability of the center of pressure, discrepancy in feet contact area, and excessive increase in plantar pressure [16,22-24]. All patients and controls underwent a static baropodometry exam that consists of standing upright, with the minimum movement possible on an electronic platform, and in 52 seconds the following parameters were obtained: maximum and average sole pressure (kgf/cm^2^), dislocation of the center of lateral and anteroposterior body gravity (as body weight percentage), and bilateral sole support surface (cm^2^).

A FootWork Analysis System (Paris, France) force platform was used that comprised 2704 capacitive sensors measuring 7.62 × 7.62 mm, that provides a stabilometric analysis, pressure discharge. The evaluation of patients consisted in the measurement of clinical postural parameters, as well as postural influence. To exclude confounding factors all patients underwent the evaluations described below, that were done by the same investigator (CWK). (i) Podal analysis: the type of feet were identified and classified as normal, curved or flat, with the last two further graded from I to III. The heel bones (calcaneus) were classified as normal, inward or outward [23]. (ii) Temporal mandibular joint: the type of bite was investigated and classified as normal, open, closed, or crossed, as well as the presence of not of bruxism. The scapula heights were measured and in case of asymmetry, the molar wedge test was performed [22]. (iii) Optical system: was investigated through ocular convergence of divergence tests, as well as the use or not of spectacles or contact lenses and the type of ocular problem. If the patient needed lenses or spectacles, the baropodometry exam was performed with them being worn, thus preventing inadequate visual information [22]. (iv) Vestibular system: patients were asked about the presence of frequent dizziness or vertigo, and if affirmative, the Fukuda test was performed. (v) Postural evaluation: the upper limb length tests were performed (rotation on the body vertical axis), and myofascial paravertebral tension through thumb test [22,23].

During the anamnesis, data related to chronic pelvic pain were collected, such as diagnosis, treatment, pain sites and its intensity. Baseline data such as social and gynecological history, as well as identification data and body mass index (kg/m^2^) (BMI) were obtained in the consultation visit. Pain score was classified according to intensity through the visual analog scale (VAS) [25]. Patients were specifically demanded about pain location by mapping the abdominal region, and situations in which it occurred. All patients were specifically asked about the previous diagnosis of endometriosis and other likely CPP causes.

### Statistics

All parameters were compared between both groups. Absolute data were analyzed with the chi-square or Fisher exact tests. Continuous variables were compared with Student-t or Mann-Whitney-U tests according to their characteristics. The SPSS 13.0 statistical package was used and data analysis was considered as statistically significant when *P *< 0.05. A sample size calculation was made considering the maximum sole pressure as primary outcome. Based in a pilot study, calculated sample size was of 25 patients in each group based on the following assumptions: (ii) prevalence of postural abnormalities of 20%, (iii) expect difference of 30% between the groups found in previous data [5], (iv) type I and II errors of 0.05 and 0.18 respectively.

## Results

The average age of the study group was of 32.5 ± 6.9 years (mean ± SD) and the control group of 28.2 ± 5.3 years (p = 0.009, t Test). The BMI for the study group was of 23 ± 3.36 and for the control group, 21.7 ± 2.3 (p = 0.08, t Test).

Mean duration of CPP in our study group was 7.3 ± 2.1 years. Table 1 demonstrates pelvic pain characteristics between the groups. As expected study group presented higher pains levels than control group. Study group presented greater number of patients with bilateral pain on hypogastric region 25 (78.2%) than control group 4 (13.3%) (p = 0.0001, chi-square). Considering the existence of associated lumbar pain, 30 (93.75%) patients in the study group presented lumbar pain compared to 21(70.0%) patients in control group (p = 0.001, chi-square).

**Table 1:** Distribution of samples characteristics (mean ± SD)

	Study group (n = 32)	Control group (n = 30)	P
Age (years)	32.5 ± 6.9	28.2 ± 5.3	0.009^1^
BMI (kg/m^2^)	23 ± 3.36	21.7 ± 2.3	0.08^1^
VAS	7.46 ± 2.14	2.2 ± 1.9	0.0001^1^
Pain - hypogastric region			
Left	7 (21.8)	2 (6.6)	0.14^2^
Bilateral	25 (78.2)	4 (13.3)	0.0001^2^
Dysmenorrea + dyspareunia +daily life activities	11 (34.4)	5 (16.6)	0.15^2^
Associated lumbar pain	30(93.7)	21(70.0)	0.001^2^

^1^T Test ^2 ^Chi-square

As shown in table 2 study and control groups presented similar averages for the maximum pressures to the left sole (3.82 ± 1.27 kgf/cm^2^; 3.87 ± 1.21 kgf/cm^2^, respectively) with no statistically significant differences (*P *= 0.87). As to the maximum sole pressure to the right, the averages found were of 2.56 ± 1.12 kgf/cm^2 ^for study group, and 2.78 ± 0.95 kgf/cm2 for control group, with no difference (*P *= 0.39).

**Table 2:** Distribution of baropodometry results between study and control groups (mean ± SD)

	Study group (n = 32)	Control Group (n = 30)	**P**^**b**^
Pressure on left foot ^a^	3.82 ± 1.27	3.78 ± 1.21	0.87
Pressure on right foot ^a^	2.56 ± 1.12	2.78 ± 0.95	0.39
Percentage of body weight displaced to the forefeet	45 ± 12.49	45.16 ± 12.08	0.95
Percentage of body weight displaced to the hindfeet	55.03 ± 11.67	55.1 ± 12.44	0.98
Percentage of body weight displaced to the left	58.59 ± 6.5	56.43 ± 5.59	0.16
Percentage of body weight displaced to the right	41.4 ± 6.5	43.56 ± 5.59	0.16

^a ^Maximum sole pressure on feet in Kgf/cm^2 b ^Student t

Study group presented an average of 45 ± 12.49 (%) of sole support on the forefeet, and the control group presented an average of 45.1 ± 12.08 (%), with no statistically significant differences (p = 0.95). In relation to the averages obtained by the study and control groups regarding sole support on hind feet, 55.03 ± 11.69 (%) and 55.1 ± 12.44 (%) respectively were found, with no difference (*P *= 0.98). Study and control groups were similar in relation to average of body weight displaced to the left [58.59 ± 6.50(%) and 56.43 ± 5.59(%) P = 0.16, respectively]. The averages of body weight displaced to the right side were of 41.40 ± 6.50 (%) and 43.56 ± 5.59 (%), for the study and control groups, respectively, presenting (*P *= 0.16) (Table 2).

According to the sole surface of feet support, the groups did not differ, with the study group presenting on the left foot support surface an average of 117.28 ± 24.84 (cm^2^) and for the control group the average was 107.52 ± 19,80 (cm^2^), (*P *= 0.97). The right foot support surface for the study group was of 120.89 ± 20.27 (cm^2^) and for the control group, 113.01 ± 19.59 (cm^2^), (*P *= 0.12). According to the postural evaluation, the groups did not differ (*P *> 0.05) - data not shown.

## Discussion

We have shown, for the first time in medical literature, that women suffering from CPP do not present differences in the baropodometric analysis when compared to healthy controls. The importance of such finding is vast, since CPP could cause postural changes by changing some muscular and even skeletical structures that would, in turn, precipitates or maintain CPP as a "cycle of pain". This data demonstrates that postural abnormalities resulting from CPP are not demonstrated by baropodometric evaluation.

In agreement with our hypothesis, some authors demonstrated that women with pelvic pain present postural abnormalities [11,12]. These women have postural changes like: (a) anterior deviation of the pelvis due to anterior leaning as a result of the anterior rotation of the iliac and the increased nutation of the sacrum; (b) increased lumbar lordosis; (c) hyperextension of the knees and anterior displacement of the gravity center in relation to the lower limbs and the pelvis itself. Often there is weakness, misbalance or muscular unbalance, tensioning and shortening of the muscular chains aggravating the toppling of the pelvis, retropulsion of the sacrum and pelvis and myofascial pain syndrome with active and latent trigger points on the iliopsoas muscles, quadratus lumborum, external rotators, and hip adductors [9]. Another elegant study showed that clinical exams revealed that patients suffering from CPP presented standard characteristics when in orthostatic position, seated, and walking, as well as lack of coordination and irregular rise in costal breathing. Many of these patterns described could be called "pelvic pain protection pattern". Therefore, the posture of patients suffering from CPP should be inherently unstable and the "pelvic pain protection pattern" considered is used as a balance strategy [5]. These authors have demonstrated nonfunctional motor and respiration patterns in CPP patients that could contribute to a vicious circle that should be take into account when planning patient treatment [5,15].

Our results show that static baropodometric evaluation is unable to detect these abnormalities. Some points can have contributed to this fact: (i) baropodometry can demonstrate postural changes, however CPP patients could have this abnormality more evident in dynamic profs and not only on static exams, (ii) there may be a postural compensatory mechanism in these patients that allows normal pressures in the feet.

We have conducted a careful study to evaluate alterations in the baropodometry exam associated to CPP. We have excluded potential confounding factors that could be associated to sole pressure changes or other parameters studied. Our research protocol included the evaluation of sensitive factors capable of affecting the posture, such and podal factor, temporal mandibular joint, ocular and vestibular system. Indeed, our control group of healthy women was meticulously chosen to be compared with the study group, and this is a very important point when evaluating patients with chronic diseases [26].

On other hand, our study has possible limitations. We could suggest that there was no difference in the baropodometry scores, since we suppose that postural changes associated to CPP are extremely subtle or compensated to be detected by this exam, which is a static evaluation [22]. This theory may be proven if we study the stabilometric phenomena in this population, since with this exam we can observe necessary body oscillations to organize the static posture within the supporting polygon. We did not show an association of the pain site with the pressure deviation in our sample. Probably due to the previous explanation, the presence of a postural compensation mechanism that corrects the posture after painful stimuli could be present in these patients, making baropodometric analysis incapable of detecting any abnormality in the sole imprint. This phenomenon could only be evaluated through stabilometry and statokinesiometry exams.

CPP causes modifications in several dimensions of patients' personal life. Studies have associated CPP with reduction in quality-of-life scores, especially in physical and psychological dimensions [1,27,18]. These aspects stress the importance pain study in all dimensions. A multidisciplinary approach is necessary to treat patients with pelvic pain.

## Conclusions

In conclusion, our data demonstrates that postural abnormalities resulting from CPP are not demonstrated by baropodometric evaluation. Other postural measures should be addressed to evaluate the complex body characteristics of pelvic pain patients. These patients would certainly benefit from comprehensive and multidisciplinary approach.

## List of Abbreviations

(CPP): Chronic Pelvic Pain; (SPSS): Statistical Package for the Social Sciences; (VAS): Visual Analogue Score.

## Competing interests

The authors declare that they have no competing interests.

## Authors' contributions

CAS, CWK, JSL conceived and designed the study. CAS and JC analyzed and interpreted the data. CWK, VKG and JSL supervised and reviewed all the statistical analysis. CAS, MA, GB and TM contributed to data collection and performed surgical procedures. All the authors contributed to write the manuscript. All the authors approved the final version of the manuscript.

## Pre-publication history

The pre-publication history for this paper can be accessed here:

http://www.biomedcentral.com/1472-6874/11/51/prepub
